# A 104-bp Structural Variation of the *ADPRHL1* Gene Is Associated With Growth Traits in Chickens

**DOI:** 10.3389/fgene.2021.691272

**Published:** 2021-08-26

**Authors:** Tong Li, Bingjie Chen, Chengjie Wei, Dan Hou, Panpan Qin, Zhenzhu Jing, Haoran Ma, Xinran Niu, Chunxiu Wang, Ruili Han, Hong Li, Xiaojun Liu, Huifen Xu, Xiangtao Kang, Zhuanjian Li

**Affiliations:** ^1^College of Animal Science and Technology, Henan Agricultural University, Zhengzhou, China; ^2^Henan Innovative Engineering Research Center of Poultry Germplasm Resource, Henan Agricultural University, Zhengzhou, China

**Keywords:** chicken, *ADPRHL1*, muscle, pseudoenzyme, structural variation

## Abstract

Analyzing marker-assisted breeding is an important method utilized in modern molecular breeding. Recent studies have determined that a large number of molecular markers appear to explain the impact of “lost heritability” on human height. Therefore, it is necessary to locate molecular marker sites in poultry and investigate the possible molecular mechanisms governing their effects. In this study, we found a 104-bp insertion/deletion polymorphism in the 5′UTR of the *ADPRHL1* gene through resequencing. In cross-designed F_2_ resource groups, the indel was significantly associated with weight at 0, 2, 4, 6, and 10 weeks and a number of other traits [carcass weight (CW), semi-evisceration weight (SEW), evisceration weight (EW), claw weight (CLW), wings weight (DWW), gizzard weight (GW), pancreas weight (PW), chest muscle weight (CMW), leg weight (LW), leg muscle weight (LMW), shedding Weight (SW), liver rate (LR), and leg muscle rate (LMR)] (*P* < 0.05). In brief, the insertion-insertion (II) genotype was significantly associated with the greatest growth traits and meat quality traits, whereas the values associated with the insertion-deletion (ID) genotype were the lowest in the F_2_ reciprocal cross chickens. The mutation sites were genotyped in 4,526 individuals from 12 different chicken breeds and cross-designed F_2_ resource groups. The II genotype is the most important genotype in commercial broilers, and the I allele frequency observed in these breeds is relatively high. Deletion mutations tend to be fixed in commercial broilers. However, there is still considerable great potential for breeding in dual-purpose chickens and commercial laying hens. A luciferase reporter assay showed that the II genotype of the *ADPRHL1* gene possessed 2.49-fold higher promoter activity than the DD genotype (*P* < 0.05). We hypothesized that this indel might affect the transcriptional activity of *ADPRHL1*, thereby affecting the growth traits of chickens. These findings may help to elucidate the function of the *ADPRHL1* gene and facilitate enhanced reproduction in the chicken industry.

## Introduction

ADP-ribosylhydrolase like-1 (ADPRHL1, encoded by the *ADPRHL1* gene) is a pseudoenzyme expressed during myocardial development in all vertebrates (Smith et al., 2020). Pseudoenzymes cannot catalyze chemical reactions, but researchers have observed that they can perform a variety of other tasks in cells (Leslie, 2013; Adrain, 2020). Accumulating evidence has shown that these pseudoenzymes play important roles in regulating inflammation and growth factor signal transduction; therefore so they play important roles in growth and development and in many diseases (Zhang et al., 2014; Adrain, 2020).

ADPRHL1 is so named because of its sequence similarity to a small group of ADP-ribosylhydrolase enzymes encoded in vertebrate genomes: ADPRH (sometimes called ARH1), ADPRHL1 (ARH2), and ADPRHL2 (ARH3). Mono-ADP-ribosylation of proteins is a post-translational modification catalyzed by ADP-ribosyltransferase and a number of bacterial toxins (Tsuge and Tsurumura, 2014). The natural protein target of ADPRH has not been fully elucidated, and while mice lacking *ADPRH* are viable, evidence obtained from this knockout indicates that ADPRH acts as an important tumor suppressor (Tang et al., 2013). In zebra fish embryos, the expression of *ADPRH* was briefly detected in the formed somatic cells, suggesting that ADP-ribosylation may be involved in skeletal muscle development (Reischauer et al., 2014). Studies on another member of the homologous gene family, ADP-ribosylhydrolase-like 2 (ADPRHL2), showed that it has a 22% amino acid sequence homology with ADPRH and can act on two distinct classes of substrates.

ADPRHL1 (Ono et al., 2006) is another member of this protein family, and the 354-amino-acid sequences of human ADPRHL1 exhibits have 46% identity to ADPRH. Interestingly, ADPRHL1 seems to lack similar enzymatic activity compared to the other two proteins (Ono et al., 2006). Pseudoenzymes similar to ADPRHL1 can be a challenge to study, but there is accumulating evidence that ADPRHL1 may be an important factor in cardiogenesis. Gene knockdown and overexpression experiments demonstrate that *ADPRHL1* is essential for heart chamber outgrowth and that alteration of *ADPRHL1* expression levels affects myofibril assembly. Elevated ADPRHL1 is associated with disarrayed myofibril patterns, contractile filaments with diverging orientations and prominent branches at the actin-Z-disc boundary (Smith et al., 2016). However, to date, whether *ADPRHL1* plays a similar role in the growth and development of poultry is unknown.

Long sequence indels longer than 50 bp are also called structural variation (SVs), which is the main mechanism of genome evolution (Kidd et al., 2010; Depristo et al., 2011). Genomic SVs seem to be more useful than SNPs to explain the diversity of human populations (Zhuo et al., 2020). Accumulating researchers have focused on the importance of SVs for animal phenotypes and diseases. For example, there has been found that SVs are associated with a variety of complex traits in dairy cows, and may potentially contribute to improving the accuracy of genomic prediction of dairy traits (Chen et al., 2021).

However, whether *ADPRHL1* gene has a similar effect in poultry and the polymorphism of *ADPRHL1* gene has not been studied. Here, a novel 104-bp SV in the 5′UTR of the *ADPRHL1* gene was employed as a genetic marker, and genotyped in an F_2_ designed full-sib resource population. The association of *ADPRHL1* gene polymorphisms with chicken growth and carcass traits was analyzed. Our dataset confirms the importance of pseudoenzymes for biological growth. The mRNA levels of the *ADPRHL1* gene were determined by qPCR to investigate the expression pattern of the gene and to confirm the deduced association between *ADPRHL1* polymorphisms and phenotypes. Our dataset elucidates the importance of pseudoenzymes for biological growth.

## Materials and Methods

### Ethics Statement

All applicable international, national, and institutional guidelines for the care and use of animals were followed. All animal experiments were performed according to the Regulations of the Chinese National Research Council (1994) and approved by the Henan Agricultural University Institutional Animal Care and Use Committee (Permit Number: 11–0085).

### Resource Populations and Traits for Association Analysis

The F_2_ resource population was constructed by cross-breeding Gushi chickens (GSs) and Anak broiler chickens (AKs). The F_1_ generation was formed through the hybridization of 24 Gushi hens with 4 Anak cocks and 12 Anak hens with 2 Gushi cocks. From this hybrid combination, individuals were selected according to phenotypic quality and heterozygosity to increase the separation of F_2_ traits, and a maximum of nine hens were randomly selected to mate with each rooster. F_2_ individuals consisted of seven families and were mated with unrelated hens (the cock was not related to the female), resulting in a total of 783 F_2_ chickens. The F_2_ population of the hybrid offspring was raised in the same environment, and free food and water were provided to the animals. At 84 days of age, each chicken was sacrificed by cervical dislocation and decapitation. The following growth characteristics were measured during this period: body weight (BW) at 0 days and 2, 4, 6, 8, 10, and 12 weeks; shank length (SL) at 0, 4, 8, and 12 weeks; and shank girth (SG), chest width (CW), body slant length (BSL), and pelvis width (PW) at 4, 8, and 12 weeks. All the detailed measuring methods were described previously (Ren et al., 2019).

Two blood samples were taken from the jugular vein during slaughter. One sample was placed in a centrifuge tube for separation of the serum and stored at −80°C. The other sample was placed in an anticoagulation tube for DNA extraction and subsequently stored at −20°C. After blood collection, 783 individuals were slaughtered to determine the carcass weights, such as carcass weight (CW), semi-evisceration weight (SEW), evisceration weight (EW), and claw weight (CLW).

To determine the presence or absence of the insertion/deletion allele at the locus of interest, DNA from 4,526 individuals was collected from blood. Samples were obtained from the F_2_ generation resource population, commercial broilers, dual-purpose chickens, commercial laying hens, and local Chinese chickens. The number of samples provided by each group was as follows: F_2_ populations (F_2_, *n* = 783), Ross 308 broilers (*n* = 192), breed 817 (817, *n* = 74), Arbor Acres broilers (AA, *n* = 552), Cobb (*n* = 192), Hubbard (*n* = 595), Gushi chicken (GS, *n* = 284), Guifei chickens (GF, *n* = 156), Wuhei green-eggshell (WH, *n* = 441), Xichuan black-bone (XC, *n* = 572), Changshun blue-eggshell (CS, *n* = 184), commercial H-line brown layers (HL, *n* = 392), and Lohmann laying hen (LM, *n* = 64).

### DNA Isolation

Genomic DNA was obtained from fresh whole-blood samples in EDTA-K2 vacutainer tubes using phenol/chloroform extraction (Huang et al., 2014). DNA quality was assessed by gel electrophoresis, and DNA quantity was assessed by optical density (OD) using a NanoDrop 2000 Spectrophotometer (Thermo Fisher Scientific, Waltham, MA, United States). DNA samples were diluted to approximately 10 ng/μl and stored at −20°C.

### Primer Design, PCR Amplification, and Genotyping

The primers used to amplify the target fragments were designed by NCBI^1^ and synthesized by Sangon Biotech Company (Shanghai, China). The primer pairs used in this study are listed in Table 1.

**TABLE 1:** Primers used for PCR amplification of the chicken *ADPRHL1* gene and mRNA transcripts.

**Gene**	**Primer, 5′–3′**	**Size, bp**	**Tm, °C**
ADPRHL1	F: CTCATCAAGAGGCACAGCCC	114/228	60
	R: AGGTGGTTGCAGTCACAGAT		
RT-ADPRHL1	F: TTCCACGGAGGAGAAAGTGC	260	60
	R: ACTCTCAGCTAATGCCTTCATCA		
GAPDH	F: GAACATCATCCCAGCGTCCA	132	60
	R: CGGCAGGTCAGGTCAACAAC		
PGL4-II/DD	F: GG**GGTACC**CCAACCTGTAGCTTCCCTCA	221/325	61
	R: C**CATATC**TGGAATTTGGCATCTCGTGT		

*Note: The underlined font refers to the sequence of the protect base. The bold words represent the restriction site.*

DNA from each individual was subjected to indel identification and genotype analysis using *ADPRHL1* primers. Sequencing was performed by Sangon Biotech Company (Shanghai, China), and the differences in the distribution of different genotypes among populations were analyzed using SPSS 24.0 software (version 24.0; Statistical Product and Service Solutions, IBM Corporation, Armonk, NY, United States). The allele frequency and genotype frequency of each mutation point were calculated using GENEPOP software,^2^ and the polymorphism information content (PIC), effective allele numbers (Ne), expected heterozygosity (He), and observed heterozygosity (Ho) were calculated simultaneously.

### RNA Isolation, cDNA Synthesis, and qPCR

To study the expression of *ADPRHL1* in different varieties, samples were taken from chickens at different stages of development for RNA experiments. A total of eight tissues (heart, liver, spleen, lung, kidney, pancreas, breast muscle, and leg muscle) were collected from GSs and AA broilers aged 1 week. Leg muscle tissues of GSs were collected at 1 day, 1 week, 6 weeks, 14 weeks, 22 weeks, and 30 weeks. Ten, 12, 14, 16, and 18 embryonic days of Gushi chicken leg muscle tissue were collected. In addition, the breast and leg tissues of broilers at embryonic ages of 10, 12, 14, 16, and 18 and at 1 day, 1 week, and 3 weeks of age were obtained from LS chickens and AA broilers, respectively.

To study the expression level of *ADPRHL1* in chickens of different genotypes, samples were taken from Arbor Acres broilers and LS chickens of different genotypes at 3 weeks old for RNA experiments. Since the DD genotype was not obtained in AA broilers, the template was collected from the liver, muscle stomach, spleen, kidney, abdominal fat, duodenum, leg muscles, and hearts of these two broilers. All tissues were immediately placed in RNAwait (Solarbio Cat#SR0020) and stored at −80°C. The samples were treated with TRIzol reagent (Takara, Otsu, Japan) following the manufacturer’s recommended protocol. The RNA concentration and integrity were estimated spectrophotometrically using a NanoDrop spectrophotometer (Thermo Fisher Scientific, Waltham, MA, United States) and verified through electrophoresis using an agarose gel. Only samples with an OD absorption ratio (OD 260 nm/OD 280 nm) between 1.9 and 2.0 and exhibiting no signs of degradation were used for further analysis. cDNA synthesis was performed using a PrimeScript RT reagent kit with gDNA Eraser (Takara).

The qPCR reaction (10 μl) contained 5 μl of 2 × SYBR Premix ExTaq (Takara, Dalian, China), 0.5 μl of each primer, and 1 μl of cDNA (approximately 100 ng/μl). qPCR conditions were as follows: 95°C for 5 min, followed by 35 cycles at 95°C for 30 s, 60°C for 30 s, and 72°C for 30 s. The reactions were performed using the QuantStudio 5 and a ProFlex^TM^ PCR instrument. The results were analyzed using the 2^–ΔΔ*CT*^ method (Schmittgen, 2008). The figures were drawn using GraphPad Prism 6 (GraphPad Software Inc., 2007, San Diego, CA, United States).

### Plasmid Construction

The allele fragment of *ADPRHL1* was amplified by polymerase chain reaction (PCR) using primers in Table 1, and cloned into a PMD18-T vector (Takara, Tokyo, Japan). To construct the luciferase reporter, the insert was released by *Eco*RV and *Kpn*I digestion and was subsequently subcloned into the luciferase reporter vector pGL4-Basic (Promega, Madison, WI, United States). Two vectors, designated the pGL4-pro1-inserted allele and pGL4-pro1-deleted allele, were constructed to test the effects of the *ADPRHL1* indel. The respective sequences of these vectors were obtained from 30 GS chickens mixed gene pools. The vector pRL-TK (Promega, Madison, WI, United States) was used as an internal reference to the luciferase reporter assay.

### Cell Culture

Human embryonic kidney 293T (HEK293T) cells were seeded in 96-well plates at a density of 5 × 10^4^ cells per well, and 100 μl growth medium was added to each well. The cells were grown in Dulbecco’s modified Eagle’s medium (DMEM) supplemented with 10% fetal bovine serum (Invitrogen) and double antibiotic (1% penicillin and streptomycin) at 37°C in a humidified 5% CO_2_ atmosphere.

### Data Analysis

The Bonferroni test was performed for multiple comparisons. Model I was used to determine growth traits, meat quality traits, and serum variables. With BW being taken as a variable, according to the effect of BW on growth traits, type II model body traits were used for analysis (Li et al., 2019, 2020; Ren et al., 2019).

Model I: Y_ijklm_ = μ + G_i_ + S_j_ + H_k_ + f_l_ + e_ijklm_

Model II: Y_ijklm_ = μ + G_i_ + S_j_ + H_k_ + f_l_ + b (W_ijklm_ −$\bar{\text{W}}$) + e_ijklm_

Our model is designed based on least squares. In these models, Y_*ijklm*_ is the observed value, μ is the overall population mean, and G_i_ is the fixed effect of the genotype (*i* = 3), including the additive and dominant effects of the gene (additive effect values are −1, 0, and 0.1 represent II, ID, and DD genotypes, respectively, while dominant effect values 1, 1, −1, and 1 represent II, ID, and DD genotypes, respectively), S_j_ is the fixed effect of gender (*j* = 2), and H_k_ is hatching. The fixed effect of (*k* = 1, 2), f_l_ is the fixed effect of family (*l* = 1, 7), e_ijklm_ stands for random error, b is the regression coefficient of BW, W_ijklm_ is the individual slaughter weight, and $\bar{W}$ is the average slaughter weight. In this study, all data are expressed as the mean ± SEM. A *P*-value < 0.05 was considered statistically significant, and the Bonferroni test was used for multiple comparisons.

## Results

### Molecular Characterization and Expression of *ADPRHL1* in Chickens

**FIGURE 1:**
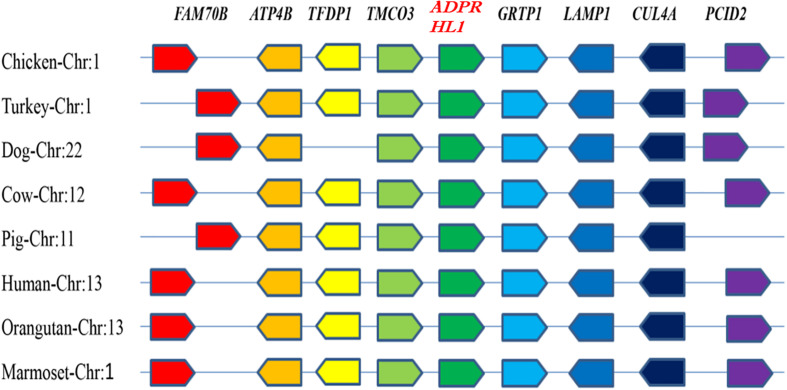
Syntenic analysis of *ADPRHL1* genes in different species. Different colors represent different genes.

The chicken *ADPRHL1* gene is located on chromosome 1 (GenBank accession number NC_006088.5), which includes two variable spliceosomes and contains eight exons that encode a protein of 1,934 amino acids. A synteny analysis was performed to examine the context of the gene in eight different species (Figure 1). The results showed that the genomic region bracketing the *ADPRHL1* gene was conserved across species and contains several common genes, including *TMCO3* (ENSGALG00000016824), *TFDP1* (ENSGALG0000001 6823), *FAM70B* (ENSGALG00000016821), *ATP4B* (ENSGA LG00000016822), *GRTP1* (ENSGALG00000016828), *LAMP1* (ENSGALG00000037697), *CUL4A* (ENSGALG00000016830), and *PCID2* (ENSGALG00000016831).

**FIGURE 2:**
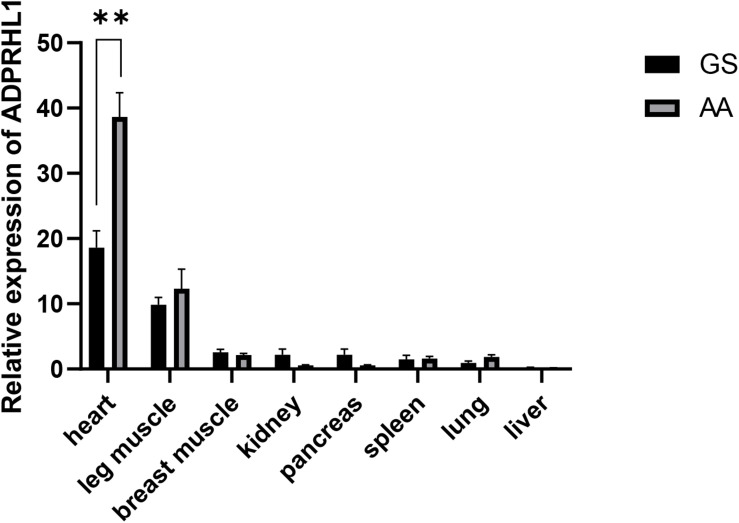
Relative expression patterns of *ADPRHL1*. Relative expression patterns of *ADPRHL1* in GS chicken and AA broiler tissues at 1 week of age. GS, Gushi chicken; AA, Arbor Acres broilers. *n* = 8. ^∗∗^*P* < 0.01.

The expression of *ADPRHL1* mRNA in various tissues of 1-week-old Gushi broilers and AA commercial broilers was analyzed. *ADPRHL1* was expressed in all the tested tissues. Notably, higher levels of expression were observed in the myocardium and leg muscles (Figure 2). The high expression of *ADPRHL1* in a variety of muscle tissues suggests a role for *ADPRHL1* in muscle-muscle formation and differentiation, which is consistent with previous findings (Smith et al., 2016).

**FIGURE 3:**
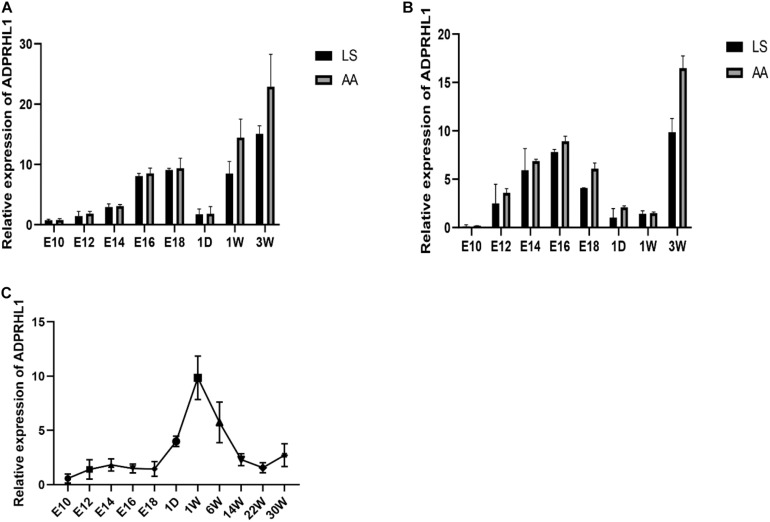
Relative expression patterns of *ADPRHL1*. **(A)** Relative expression patterns of *ADPRHL1* in the leg muscle in LS and AA chickens tissues at 10, 12, 14, 16, and 18 embryo and 1 day, 1 week, and 3 weeks of age. LS, Lushi chicken; AA, Arbor Acres broilers. *n* = 6. **(B)** Relative expression patterns of ADPRHL1 in the breast muscle in LS and AA chickens tissues at 10, 12, 14, 16, and 18 embryonic days and 1 day, 1 week, and 3 weeks of age. LS, Lushi chicken; AA, Arbor Acres broiler; *n* = 6. **(C)** Relative expression patterns of *ADPRHL1* in the leg muscle in GS chickens tissues at 10, 12, 14, 16, and 18 embryo and 1 day, 1 week, 6 weeks, 14 weeks, 22 weeks, and 30 weeks of age. GS, Gushi chicken; *n* = 6.

Expression of *ADPRHL1* gene in breast and leg muscle tissues was studied at embryonic day of 10, 12, 14, 16, and 18 and at 1 day, 1 week, and 3 weeks of age in LS chickens and AA broilers. The expression of *ADPRHL1* in breast muscle and leg muscle showed a trend of first increasing, then decreasing and then increasing at successive developmental stages. Specifically, the expression level was highest at approximately 16–18 days of embryonic life, decreased rapidly after birth, and then increased rapidly with developmental stages to reach its peak at 3 weeks of age (Figures 3A,B). The expression level of *ADPRHL1* in AA broilers was significantly higher than that in LS broilers at 3 weeks of age (*P* < 0.05). Notably, the expression of *ADPRHL1* in breast muscle did not change significantly at 1 day of age and 1 week of age, which was different from the expression observed in leg muscle. To examine the cause of this difference, we analyzed the expression level of *ADPRHL1* in the leg muscle of GS chickens. The results showed that the expression level of *ADPRHL1* in GS chicken leg muscle reached the highest level at 1 week, decreased decline from 1 to 14 weeks, and remained largely stable from 14 to 30 weeks (Figure 3C).

### Identification of Genetic Variants Correlated With *ADPRHL1* Expression

**FIGURE 4:**
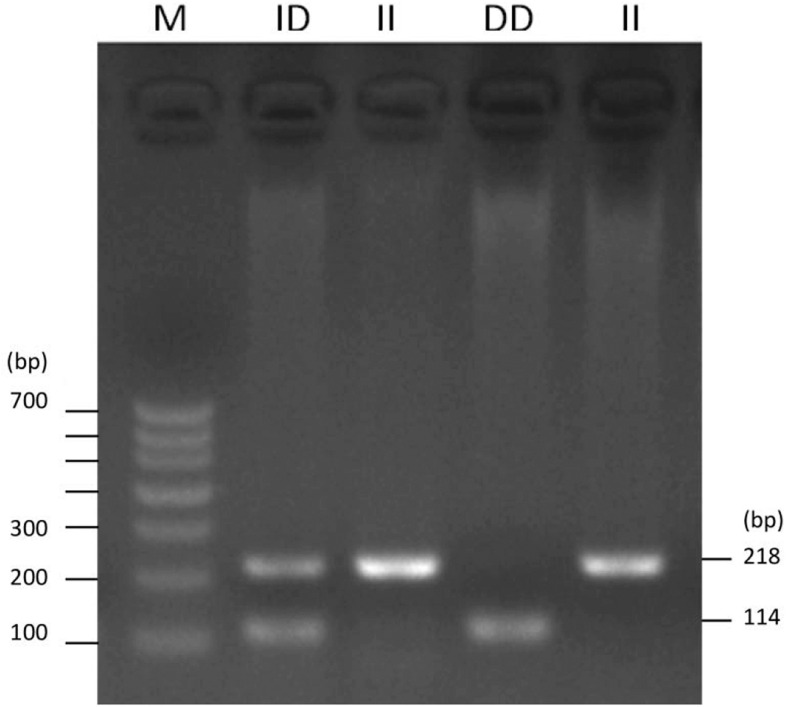
Agarose gel electrophoresis pattern for the *ADPRHL1* 104-bp indel polymorphism.

To identify the potential factors affecting the expression of *ADPRHL1*, the sequence mutation of *ADPRHL1* gene was studied. A new 104-bp deletion (GenBank accession number NC_006088.5, Chr1:138752529–138752632) mutation was detected in the 5′UTR of the *ADPRHL1* gene by whole-genome resequencing. Indel polymorphisms were genotyped by PCR amplification of the region and electrophoresis of products was performed in a 2.0% agarose gel. Three possible genotypes were identified, designated II (218 bp), ID (218 bp and 114 bp), and DD (114 bp) (Figure 4).

**FIGURE 5:**
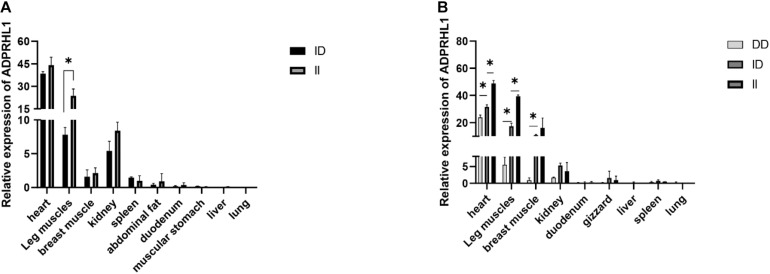
Relative expression patterns of *ADPRHL1*. **(A)** Expression patterns of different genotypes of *ADPRHL1* in Arbor Acres broiler at 1 week of age. ID, *n* = 3; DD, *n* = 5. **(B)** Expression patterns of different genotypes of *ADPRHL1* in Lushi chicken at 3 weeks of age. DD, *n* = 3; ID, *n* = 3; DD, *n* = 3. Error bars represent the SEM. ^∗^*P* < 0.05.

Table 1 details the PCR primers related to the locus. The results of qPCR showed that there were significant differences in the expression levels between ID and DD genotypes in 3-week-old Lushi chickens and 1-week-old AA broilers (no DD genotypes were found in AA broilers in our population) (*P* < 0.05, Figure 5).

These results suggest that the 104-bp indel locus affects the expression of *ADPRHL1* and may influence the growth traits of chickens. It was therefore important to examine the relationship between the 104-bp indel locus and growth traits in a larger chicken population.

### Genetic Parameters of the 104-bp Indel Locus

To examine the relationship between the 104-indel locus and growth traits in chickens, allele frequencies and other genetic parameters associated with the *ADPRHL1* Indel locus were calculated for 4,526 individuals in the study. This analysis included F_2_ generation resource populations, dual-purpose chickens (GS, GF, WH, XC, and CS), commercial laying hens (HL and LM) and commercial broilers (Ross 308, 817, Hubbard, Cobb, and AA). The frequency of genotype II in commercial broilers (Ross 308/67%, 817/70%, AA/99%, Cobb/74%, and Hubbard/77%) was notably greater than that in any other breed (F_2_/43%, GS/37%, GF/25%, WH/46%, XC/47%, CS/39%, HL/22%, and LM/42%) in our study. The frequency of the DD genotype was lower in all varieties, except type GF, and it was highly rare in commercial broilers (Ross 308/4%, 817/0%, AA/0%, Cobb/1%, and Hubbard/0%). Finally, the frequency of the I alleles in commercial broilers (Ross 308/81.2%, 817/85.1%, AA/99.7%, Cobb/86.7%, and Hubbard/88.4%) was higher than that in other breeds (F_2_/70.1%, GS/68.0%, GF/48.7%, WH/67.6%, XC/69.5%, CS/62.5%, HL/55.6%, and LM/64.8%) (Table 2).

**TABLE 2:** Genetic parameters of 104-bp locus within *ADPRHL1* gene in F_2_ and other 12 chicken breeds.

	Breeds/*n*	Genotypic and allelic frequencies					Ho	He	Ne	PIC	*P*-value, HWE
		DD	ID	II	D	I					
F_2_ generation resource population	F_2_/783	0.03	0.54	0.43	0.299	0.701	0.54	0.42	1.723	0.332	0.000
Commercial broilers	Ross 308/192	0.04	0.29	0.67	0.188	0.812	0.29	0.31	1.438	0.258	0.554
	817/74	0.00	0.30	0.70	0.149	0.851	0.30	0.25	1.339	0.221	0.133
	AA/552	0.00	0.01	0.99	0.003	0.997	0.01	0.01	1.005	0.005	0.949
	Cobb/237	0.01	0.25	0.74	0.133	0.867	0.25	0.23	1.299	0.204	0.076
	Hubbard/595	0.00	0.23	0.77	0.116	0.884	0.23	0.21	1.258	0.184	0.001
Dual-purpose chickens	GS/284	0.01	0.62	0.37	0.320	0.680	0.62	0.44	1.771	0.341	1.000
	GF/156	0.28	0.47	0.25	0.513	0.487	0.47	0.50	1.999	0.375	0.527
	WH/441	0.11	0.43	0.46	0.324	0.676	0.43	0.44	1.78	0.342	0.723
	XC/572	0.08	0.46	0.47	0.305	0.695	0.46	0.42	1.736	0.334	0.044
	CS/184	0.14	0.48	0.39	0.375	0.625	0.48	0.47	1.882	0.359	1.000
Commercial laying hens	HL/392	0.11	0.67	0.22	0.444	0.556	0.67	0.49	1.975	0.372	0.000
	LM/64	0.13	0.45	0.42	0.352	0.648	0.45	0.46	1.838	0.352	0.961

*F_2_, F_2_ resource population; GS, Gushi chicken; GF, Guifei chicken; WH, Wuhei chicken; XC, Xichuan black-bone chicken; CS, Changshun green-shell chicken; Ho, observed heterozygosity; He, expected heterozygosity; Ne, effective allele numbers; PIC, polymorphism information content; *P*-value (HWE), *P*-value of Hardy–Weinberg equilibrium. PIC > 0.50 represents high polymorphism, while 0.25 < PIC < 0.50 represents moderate polymorphism, and PIC < 0.25 represents low polymorphism.*

These results suggested that genotype II was the dominant genotype and has been highly selected in commercial broilers. The genetic indices (Ho, He, Ne, and PIC) for these 13 chicken populations are presented in Table 2. Ho, He, Ne, and Pic are not only indicators to measure allelic polymorphisms and the degree of gene mutation, but also indicators of genetic variation within a population. In commercial broilers, the observed heterozygosity was in the range of 0.01–0.03 and the expected heterozygosity was in the range of 0.01–0.31. In addition, all the breeds exhibited low polymorphism except Ross 308 in commercial broilers. In the F_2_ generation resource population, dual-purpose chickens and commercial laying hens, the observed heterozygosity was in the range of 0.43–0.67 and the expected heterozygosity was in the range of 0.42–0.50. Moreover, the F_2_ generation resource population, dual-purpose chickens and commercial laying hens showed moderate polymorphism, indicating that they have greater genetic variation and selection potential. These results suggested that commercial broiler breeds (especially Arbor Acres broilers) might have experienced great pressure of artificial selection.

### Association Analysis of 104-bp Insertion/Deletion of the *ADPRHL1* Gene With Growth, Carcass, Meat Quality Traits, and Biochemical Variables

The results of the correlation analysis show that the 104-bp indel polymorphism was significantly associated with BW at 4, 6, and 10 weeks of age and with body size indices, including 0-week SL and 4-week BSL (*P* < 0.05), in addition to exhibiting a highly significant correlation with BW at 0 and 2 weeks (*P* < 0.01) (Table 3). The carcass traits were significantly associated with CW, SEW, EW, CLW, DWW, GW, PW, CMW, LW, LMW, and SW (*P* < 0.05); Moreover, there were significant associations between this 104-bp indel polymorphism and meat quality traits (Table 4). The results of the serum variable association analysis further showed that the indel was significantly associated with alanine transaminase and lactate dehydrogenase (*P* < 0.05) (Table 5).

**TABLE 3:** Association of the 104-bp locus with growth traits in an F_2_ population at 84 days of age.

Growth traits	DD (*n* = 23)	ID (*n* = 423)	II (*n* = 337)	*P*-value
0-week weight (g)	31.54 ± 0.54^ab^	30.368 ± 0.13^a^	31.012 ± 0.15^b^	0.002
2-week weight (g)	124.683 ± 3.71^ab^	120.716 ± 0.92^a^	125.162 ± 1.05^b^	0.005
4-week weight (g)	323.474 ± 9.65^ab^	318.585 ± 2.23^a^	327.264 ± 2.55^b^	0.037
6-week weight (g)	541.638 ± 17.47^ab^	558.166 ± 4.21^a^	575.141 ± 4.82^b^	0.012
10-week weight (g)	1088.957 ± 32.13^ab^	1104.007 ± 7.77^a^	1133.963 ± 8.84^b^	0.027
0-week shank length (cm)	2.603 ± 0.02	2.568 ± 0.01	2.584 ± 0.01	0.026
4-week body slant length (cm)	11.526 ± 0.16^ab^	11.337 ± 0.04^a^	11.479 ± 0.04^b^	0.034

*SE, standard error of the mean. Means with different superscripts indicate significant differences: different lowercase letter indicates *P* < 0.05; and the same letters indicate no difference (*P* > 0.05).*

**TABLE 4:** Association of the 104-bp locus with carcass traits in an F_2_ population at 84 days of age.

Traits	DD (*n* = 23)	ID (*n* = 423)	II (*n* = 337)	*P*-value
CW (g)	1209.287 ± 33.91^ab^	1204.192 ± 8.30^a^	1238.255 ± 9.45^b^	0.024
SEW (g)	1094.707 ± 32.236^ab^	1089.126 ± 7.92^a^	1124.588 ± 9.05^b^	0.012
EW (g)	915.842 ± 28.45^ab^	909.558 ± 6.87^a^	940.244 ± 7.82^b^	0.013
CLW (g)	57.397 ± 2.00^ab^	57.966 ± 0.49^a^	60.013 ± 0.56^b^	0.017
WW (g)	121.871 ± 3.74^ab^	121.142 ± 0.92^a^	124.962 ± 1.04^b^	0.022
GW (g)	27.003 ± 0.92^ab^	27.502 ± 0.23^a^	28.725 ± 0.26^b^	0.001
PW (g)	3.484 ± 0.14^ab^	3.315 ± 0.03^a^	3.437 ± 0.04^b^	0.036
CMW (g)	71.317 ± 2.97^ab^	69.09 ± 0.73^a^	72.527 ± 0.83^b^	0.008
LW (g)	147.419 ± 4.69^ab^	148.274 ± 1.15^a^	152.652 ± 1.31^b^	0.036
LMW (g)	96.548 ± 3.47^ab^	98.228 ± 0.86^a^	101.945 ± 0.98^b^	0.011
SW (g)	1184.227 ± 33.45^ab^	1178.477 ± 8.20^a^	1211.151 ± 9.32^b^	0.03
LR (%)	2.087 ± 0.06^ab^	2.179 ± 0.02^a^	2.108 ± 0.02^b^	0.006
LMR (%)	20.894 ± 0.30	21.48 ± 0.07	21.639 ± 0.08	0.039
Pectoral fullness (%)	0.924 ± 0.004^a^	0.917 ± 0.001^ab^	0.914 ± 0.001^b^	0.013
Breast muscle water holding capacity (%)	14.747 ± 0.85	16.728 ± 0.21	16.055 ± 0.23	0.016

*CW, carcass weight; SEW, semi-evisceration weight; EW, evisceration weight; CLW, claw weight; DWW, wings weight; GW, gizzard weight; PW, pancreas weight; CMW, Chest muscle weight; LW, Leg weight; LMW, leg muscle weight; SW, Shedding Weight; LR, liver rate; LMR, leg muscle rate; SE, standard error of the mean. Different lowercase letters of the means superscript show significant differences (*P* < 0.05), the same letters show no difference (*P* > 0.05).*

**TABLE 5:** Association of the 104-bp locus with serum variables in an F_2_ population at 86 days of age.

Serum variables	DD (*n* = 23)	ID (*n* = 423)	II (*n* = 337)	*P*-value
Alanine transaminase	2.593 ± 0.45^a^	1.648 ± 0.11^b^	1.916 ± 0.12^b^	0.046
Lactate dehydrogenase	2641.470 ± 102.90^ab^	2734.110 ± 23.62^a^	2845.666 ± 26.38^b^	0.003

*SE, standard error of the mean. Means with different superscripts indicate significant differences: different lowercase letter indicates *P* < 0.05; and the same letters indicate no difference (*P* > 0.05).*

### Identification of 104-bp Insertion/Deletion of *ADPRHL1* Transcriptional Activity

**FIGURE 6:**
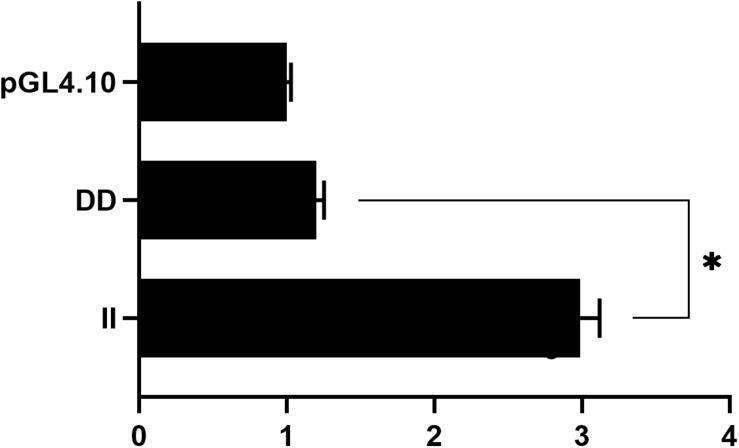
Relative luciferase activity detection of *ADPRHL1* gene promoter. Luciferase activity in HEK293T cells transfected with recombinant plasmids containing serial promoter fragments (pGL4-II, pGL4-DD) of the *ADPRHL1* promoter. The backbone vector pGL4.10 was used as a negative control; Each experiment was repeated at least five times. The data were the mean ± standard error (S.E.) of the normalized luciferase activity (^∗^*P* < 0.05).

To elucidate the molecular mechanisms associated with 104-bp indel at the cellular level, we attempted to identify changes in promoter activity across different genotypes. Pooled genomic DNA from 30 GS chickens was utilized as a template, and various genotypes were obtained by PCR using a pair of specific primers (Table 1). The transcriptional activity of the different genotypes was subsequently measured in HEK293T cells using a dual luciferase reporter analysis system. Genotype II exhibited a 2.49-fold higher activity than the genotype DD (*P* < 0.05) (Figure 6).

## Discussion

*ADPRHL1* is only widely expressed in vertebrates, suggesting that it may be a driving force in evolution (Smith et al., 2016). In addition, it was previously observed that ADPRHL1 expresses one of the mRNAs induced cardiac differentiation of human embryonic stem cells *in vitro* (Beqqali et al., 2006). As a pseudoenzyme-expressing gene, the specific mechanism of *ADPRHL1* in biological growth and development has not been determined. In our study, we first indirectly suggested the important role of *ADPRHL1* in chicken heart, leg muscle, chest muscle, and other tissues, by analyzing the expression of this gene in different tissues at different times. Notably, *ADPRHL1* appears to play a role at different times in different organizations. Previous studies have suggested that *ADPRHL1* plays a direct role in the modification of Z-disc and actin dynamics (Smith et al., 2020). In our study, *ADPRHL1* has higher expression in the breast muscle and heart, and we surmise that it may have a positive effect on muscle development. This may be on account of Adprhl1 plays a similar role in chickens. We believe that *ADPRHL1* is involved in the development of chicken embryos in the middle and late stages of development and primarily mainly functions between 0 and 6 weeks after birth.

Many studies have elucidated the effects of other 5′UTR indel mutations on animal growth and development (Glanzmann et al., 2014). For example, the 18-bp indel in the porcine *SOX9* 5′-UTR is of functional importance and may therefore indeed be a causative variation in *SOX9* associated traits (Brenig et al., 2015). Genome SVs is a major source of genetic and phenotypic variation and has received increasing attention (Liu et al., 2021). Some researchers have made some progress through the role of SVs in evolution and the mechanism of SVs in promoting pig phenotypic variation (Du et al., 2021). This evidence supports the importance of 5′UTR for large indel. We first discovered a novel 104-bp SV in 5′UTR of the *ADPRHL1* gene in whole-genome resequencing of thirty GS chickens and verified it in thirteen populations. In cross-designed F_2_ resource groups, the indel was significantly associated with many growth traits, carcass traits and serum variables in our study (Tables 3–5). The results indicate the importance of this 104-bp indel for the growth and development of chickens. In addition, we further studied the genetic diversity of the mutation and characterized its genetic characteristics.

Allele frequencies are a reflection of genetic diversity between populations and may indicate genetic drift or the introduction of new mutations (Jia et al., 2016). Artificial selection has an important influence on the development of varieties and commodity populations and determines the amount and distribution of genetic variation during domestication. Notably, although genotype II is dominant in commercial broiler flocks, the gap between genotypes II and DD is considerably smaller in dual-purpose and commercial laying hens. Moreover, dual-purpose chickens and commercial laying hens showed moderate polymorphism, which indicated that they possessed greater genetic variation and selection potential than commercial broilers. Notably, Ross 308 chickens also showed moderate polymorphism, which indicated that the Ross 308 chickens still had room for breeding.

Growth and development are important processes that affect different body parts of all animals in different life stages, and are affected by many factors, including genetics, nutrient absorption and environmental conditions (Hui et al., 2020; De Robertis and Gurdon, 2021; Liu et al., 2021). We found that a significant association between 104-bp indel and growth characteristics occurred in the early stage of chicken growth. Consistent with this result, the expression of the *ADPRHL1* gene with genotype II was significantly higher in the leg muscle, breast muscle, and myocardium of 3-week-old LS chickens than that in those with genotypes ID and DD. Although the DD genotype was not observed in large numbers of AA broilers, the expression of the *ADPRHL1* gene with genotype II was significantly higher in the leg muscle of 1-week-old AA broilers than in that of the ID chickens. There was no significant difference in the expression of these two genotypes in the breast muscle of 1-week-old AA broilers, which might be related to the low expression level of *ADPRHL1* in the breast muscle of 1-week-old. Nevertheless, differences in the expression levels of *ADPRHL1* with different polymorphisms in various muscle tissues demonstrate the possibility that this indel is related to muscle development. In addition to the above description, this locus was significantly associated with such traits as PW, CW, and GW (*P* < 0.05). These results indicate that the *ADPRHL1* gene plays an important role in muscle development. Further studies should consider the role of its regulatory network and components in the development of skeletal muscle in chickens.

## Data Availability Statement

The original contributions presented in the study are included in the article/supplementary material, further inquiries can be directed to the corresponding author/s.

## Ethics Statement

The animal study was reviewed and approved by the Institutional Animal Care and Use Committee of Henan Agricultural University.

## Author Contributions

TL performed the research, analyzed the data, and wrote the manuscript. BC, CJW, DH, and PQ analyzed the data and involved in the study design. HM, XN, ZJ, and CXW performed the statistical analysis. RH, HL, XL, HX, and XK were involved in the design of the study. ZL conceived the study and involved in its design and coordination. All authors contributed to manuscript revision, read, and approved the submitted version.

## Conflict of Interest

The authors declare that the research was conducted in the absence of any commercial or financial relationships that could be construed as a potential conflict of interest.

## Publisher’s Note

All claims expressed in this article are solely those of the authors and do not necessarily represent those of their affiliated organizations, or those of the publisher, the editors and the reviewers. Any product that may be evaluated in this article, or claim that may be made by its manufacturer, is not guaranteed or endorsed by the publisher.

## Footnotes

1
http://www.ncbi.nlm.nih.gov/tools/primer-blast/

2
http://genepop.curtin.edu.au/
